# Elevated Level of Serum Neurotrophin-4, but Not of Brain-Derived Neurotrophic Factor, in Patients with Chronic Kidney Disease-Associated Pruritus

**DOI:** 10.3390/jcm11216292

**Published:** 2022-10-26

**Authors:** Kamila Wala-Zielińska, Karolina Świerczyńska-Mróz, Piotr K. Krajewski, Danuta Nowicka-Suszko, Magdalena Krajewska, Jacek C. Szepietowski

**Affiliations:** 1 Department of Dermatology, Venereology and Allergology, Wroclaw Medical University, 50-368 Wroclaw, Poland; 2 Department of Nephrology and Transplantation Medicine, Wroclaw Medical University, 50-556 Wroclaw, Poland

**Keywords:** neurotrophin-4, brain-derived neurotrophic factor, pruritus, chronic itch, chronic kidney disease, chronic kidney disease-associated pruritus

## Abstract

Chronic kidney disease-associated pruritus (CKD-aP) is a bothersome condition that occurs in patients with advanced chronic kidney disease (CKD) and severely reduces their quality of life. Recently, much research has focused on the search for markers that are involved in the pathogenesis of CKD-aP and may become a therapeutic target. One of the suggested hypotheses is the increased activation of sensory neurons by molecules such as neurotrophins (NTs). An increased serum concentration of NTs has been demonstrated in pruritic patients, which may suggest their involvement in the pathogenesis of itch. The purpose of this study is to assess the serum concentration of neurotrophin-4 (NT-4) and brain-derived neurotrophic factor (BDNF) in hemodialysis patients. The study enrolled 126 patients undergoing dialysis. Participants were divided into 2 groups: with and without CKD-aP. NRS scale was used to evaluate itch severity. Serum levels of NT-4 and BDNF have been assessed using ELISA. The results showed a significantly higher level of NT-4 in the group with pruritus. No significant difference was reported in the serum level of BDNF between the two groups of patients. There was also no correlation between serum NT-4 nor BDNF levels and the severity of pruritus. In summary, NT-4 may play an important role in the pathophysiology of pruritus in dialysis patients. More research is needed to understand the exact mechanism by which NTs influence the pathogenesis of CKD-aP.

## 1. Introduction

Chronic itch (CI) is an inconvenient sensation on the skin causing a constant urge to scratch, that lasts more than 6 weeks and remains a serious therapeutic problem for both patients and physicians [1]. It is one of the primary symptoms in dermatological diseases, but it can also accompany systemic disorders such as cholestasis, hematological neoplasms, and advanced chronic kidney disease [1,2]. Depending on the underlying disease, the mechanism responsible for the perception of pruritus is different, but most often the etiology is multifactorial [2]. Chronic kidney disease-associated pruritus (CKD-aP) is defined as a burdensome condition affecting mainly patients with advanced stages of kidney disease, the occurrence of which is associated with worse medical outcomes and higher mortality [3]. According to the Dialysis Outcomes and Practice Patterns Study report, the prevalence of at least moderate CKD-aP in hemodialysis patients is 37% [4]. Despite the growing awareness of CKD-aP, the condition is still often underdiagnosed [5]. The literature mentions numerous hypotheses concerning the pathogenesis of CI in this group of patients, however, its etiology remains unclear. The suggested causes include the influence of uremic toxins, disbalance in calcium and phosphorous metabolism, hyperparathyroidism, xerosis, imbalance of opioid transmission as well as dysfunction of the neuronal and immune systems [5,6,7,8,9]. It is supposed that the interaction between the skin and the peripheral nervous system, which conducts afferent impulses to the central nervous system, is of great importance [10]. More and more itch mediators are known that can stimulate the nerve endings in the dermis. This mechanism involves skin cells, which actively participate in the synthesis and release of neurotransmitters, cytokines, cannabinoids, endogenous opioids, and other factors that activate receptors on peripheral nerve endings [2]. Importantly, the pathophysiology of CI most often involves multiple interacting mediators rather than a single disease-specific stimulant [2]. Due to the unknown cause of CKD-aP, effective therapy for this condition is a major challenge. Moreover, antihistamines commonly used in the treatment of chronic itch do not bring significant improvement in patients with CKD-aP [11]. Current methods include UVB phototherapy, topical treatment with capsaicin, 10% urea, or tacrolimus, as well as moisturizing therapy to maintain proper skin hydration. Systemic drugs such as pregabalin, gabapentin, sertraline, and opioid agonists, including recently approved difelikefalin have also been shown to be effective [1,6,12,13]. However, considering the fact that dialysis patients often suffer from moderate to severe pruritus, current treatment options for CKD-aP are still insufficient [11].

**Figure 1:**
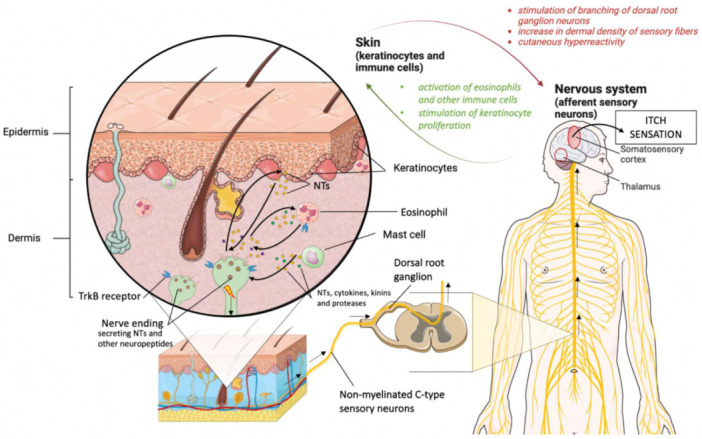
Possible pathomechanism of pruritus involving neurotrophins (NTs). Keratinocytes and skin immune cells produce NTs that activate receptors located on the cell membrane of nerve endings. The stimulated sensory neurons conduct impulses through the dorsal root ganglion to the somatosensory cortex, leading to an itching sensation. Simultaneously, the same molecules produced by non-neuronal skin cells lead also to an increase in skin density of sensory fibers and skin hyperreactivity. Moreover, nerve cells located in the skin have the ability to produce neuropeptides, including NTs, which in turn influence the proliferation of keratinocytes and the activation of immune cells, and further enhance mutual stimulation between neurons and non-neuronal skin cells. This figure was created using Servier Medical Art under a CC 3.0 license (https://smart.servier.com/, accessed on 23 September 2022) [27].

Neurotrophins (NTs) are growth factors that ensure the proper functioning of neurons. Both neurotrophin-4 (NT-4) and brain-derived neurotrophic factor (BDNF) are proteins belonging to the NTs family, responsible for the proliferation, maturation, and differentiation of nerve cells [14]. They also provide increased plasticity of neurons and their resistance to damage, and inhibit pathways that promote apoptosis, thus allowing nerve cells to survive under conditions of increased oxidative stress [14,15]. NT-4 and BDNF share common receptors and act on the tyrosine kinase B receptor (TrkB) with low affinity on the p75 neurotrophin receptor [16]. Reduced BDNF concentration in the central nervous system has been observed in neurodegenerative diseases such as Parkinson’s disease and multiple sclerosis [17]. In addition to the central and peripheral nervous systems, receptors for these proteins are expressed and have important functions in other tissues and organs such as the heart, lungs, kidneys, muscles, and skin [18]. The role of neurotrophins in the proper functioning of the skin and the pathophysiology of skin diseases is being constantly studied. It has been found that the nerve growth factors found in the skin promote the proliferation of keratinocytes, regulate hair growth, and the survival of melanocytes [10]. The involvement of these factors has also been noticed in pathological conditions of the skin with inflammation, such as psoriasis or atopic dermatitis as well as in melanoma [10,19]. Moreover, reports are suggesting the involvement of NTs in the pathogenesis of pruritus [20,21]. NTs are suspected to be involved in the interactions between keratinocytes, cells of the immune system, mainly eosinophils, and non-myelinated C-type sensory neurons responsible for the conduction of impulses that trigger the itching sensation [22,23]. In a study by Guseva et al. [24] stimulated eosinophils and neuronal projections have been observed to secrete NTs. Similarly, the neurotrophin receptor, TrkB, is expressed by both eosinophils and sensory nerve fibers [24]. Although functional TrkB has not been demonstrated on keratinocytes, NTs most likely act on these cells through the p75 neurotrophin receptor [16]. Moreover, it has been shown that NTs, such as BDNF, lead to the branching of dorsal root ganglion neurons and increase the skin density of the sensory fibers [24]. Nerve endings also release neuropeptides, which in turn bind to specific receptors on the surface of non-neuronal skin cells and lead to their activation. A branched network of interactions is formed, which further enhances the production of pruritogens [24,25]. Subsequently, neuronal projections activated by various mediators such as cytokines, NTs, kinins, and proteases, secreted by intracutaneous cells, conduct impulses through the posterior roots to the spinal cord. Then, via the spinothalamicus tract, they reach the thalamus, from where impulses are transmitted to specific areas of the brain [22]. The suggested mechanism of action of NTs in the pathogenesis of pruritus is illustrated in Figure 1.

Agarwal et al. [5] suggested that CKD-aP associated with nervous system dysfunction could be triggered in various ways, either by cortical activation by centrally acting mediators, by a nerve impulse inhibition defect, or by hyperactivation of peripheral sensory neurons [5]. Interestingly, indirect immunohistochemistry has revealed an abnormal pattern of skin innervation in patients undergoing hemodialysis. In addition, increased growth of nerve fibers throughout the epidermis was noticed [26]. Taking into account the above-mentioned research results, the most likely mechanism of action of NTs in CKD-aP seems to be related to the hypersensitivity of sensory neurons, however, further research is required to confirm this hypothesis.

The aim of the study was to assess the concentration of NT-4 and BDNF in the serum of patients undergoing hemodialysis, with and without pruritus, and to verify whether there is a difference in the level of neurotrophins between these two groups of patients. Furthermore, correlations between the concentration of NT-4 or BDNF and the severity of pruritus were determined.

## 2. Materials and Methods

### 2.1. Participants and Study Design

The study was conducted between November 2020 and April 2021. 126 hemodialysis patients from the Department of Nephrology and Transplantation Medicine at the University Hospital in Wroclaw, Poland, and the Dialysis Unit at the University Hospital in Opole, Poland were enrolled in the study. Inclusion criteria were as follows: patients over 18 years of age receiving hemodialysis 2 or 3 times a week for at least 3 months, who signed the patient’s informed consent. Dialysis patients with other chronic conditions that may cause itching were not included in the study. Moreover, additional exclusion criteria were antipruritic therapy, mental disorders, and lack of informed consent.

Basic demographic and clinical data (gender, age, cause of renal failure, duration of hemodialysis, type of vascular access, and presence of pruritus) was collected. This research received ethical approval from the Wroclaw Medical University Ethics Committee (Consent no. 26/202, date: 29 January 2021). All patients enrolled in this study have provided their informed consent.

### 2.2. Laboratory Tests

A total of 126 blood samples were taken from patients immediately (5–10 min) prior to the dialysis session. The time of blood sampling was the same for all participants. The blood samples were then centrifuged at 3000 rpm for 15 min, and the serum was stored at −80 °C until further tests were performed. Subsequently, the samples were spread out in 96-well plates. An enzyme-linked immunosorbent assay was performed according to the manufacturer’s instructions using the ELISA Kits to assess the serum level of NT-4 (Nori Human Neurotrophin 4 ELISA Kit, GR111502, Genorise Scientific, Inc., Pennsylvania, PA, USA) and BDNF (Nori Human BDNF ELISA Kit, GR111085, Genorise Scientific, Inc., Pennsylvania, PA, USA). Absorbance was measured using an EPOCH multiplate reader (BioTEK^®^ Instruments, Inc., Winooski, VT, USA) at a wavelength of 450 nm. Serum levels of NT-4 and BDNF were expressed in pg/mL.

### 2.3. Pruritus Assessment

In this study, the numerical rating scale (NRS) was used to assess the severity of pruritus in patients with CKD-aP, which is considered an easy and reliable tool for assessing the severity of pruritus. Patients were asked to rate the intensity of itching they experienced in the past 3 days on a scale of 0 to 10 points. Depending on the results, the severity of pruritus was divided into mild (NRS score < 3 points), moderate (NRS score 3–6 points), severe (NRS score 7–9 points), and very severe (NRS score > 9 points) [28]. Patients also completed the validated Polish version of the UP-Dial questionnaire [29]. This scale, dedicated to patients with CKD-aP undergoing dialysis, consists of 14 items assessing the severity of pruritus, but also its impact on various spheres of life, including skin changes caused by itching, the severity, frequency and distribution of pruritus, and the impact on psychosocial life and sleep quality [29]. Additionally, the quality of life was assessed using the ItchyQoL questionnaire (Figure S1). It is a tool designed for patients with pruritus, assessing 3 domains: symptoms, functions, and emotions. The questionnaire consists of 22 items, each scored from 1 to 5 points [30].

### 2.4. Statistical Analysis

The IBM SPSS Statistics v. 26 software (SPSS Inc., Chicago, IL, USA) was used for the statistical analysis of the results obtained in the study. Initially, all data was checked for normal or abnormal distribution. For quantitative data analysis, the Mann–Whitney U test and Pearson’s or Spearman’s correlations were used. Differences in NT-4 and BDNF between various pruritus severity groups were assessed using the Kruskal-Wallis test with Bonferroni correction. Assessment of qualitative results was evaluated with the use of the chi-squared test. Data were expressed as a minimum, maximum, mean ± SD, median, first and third quantiles with *p* < 0.05 being considered statistically significant.

## 3. Results

### 3.1. Baseline Characteristics of the Subjects

There were 61 (48.4%) men and 65 (51.6%) women among the respondents. The average age of the patients was 62.5 ± 15.8 years. The most common causes of renal failure among all participants were glomerulonephritis in 25 patients (19.8%) and diabetic nephropathy in 24 patients (19.0%).

Based on the presence of pruritus, participants were divided into 2 groups. The pruritic group (group A) included 62 participants and the non-pruritic group (group B) had 64 participants. The groups were similar in terms of gender and age, with a mean age of 61.1 ± 15.9 years and 63.9 ± 15.6 years, respectively.

The baseline characteristics of the groups analyzed in this study are presented in Table 1.

### 3.2. Serum Levels of NT-4 and BDNF in Itchy and Non-Itchy Patients

**Figure 2:**
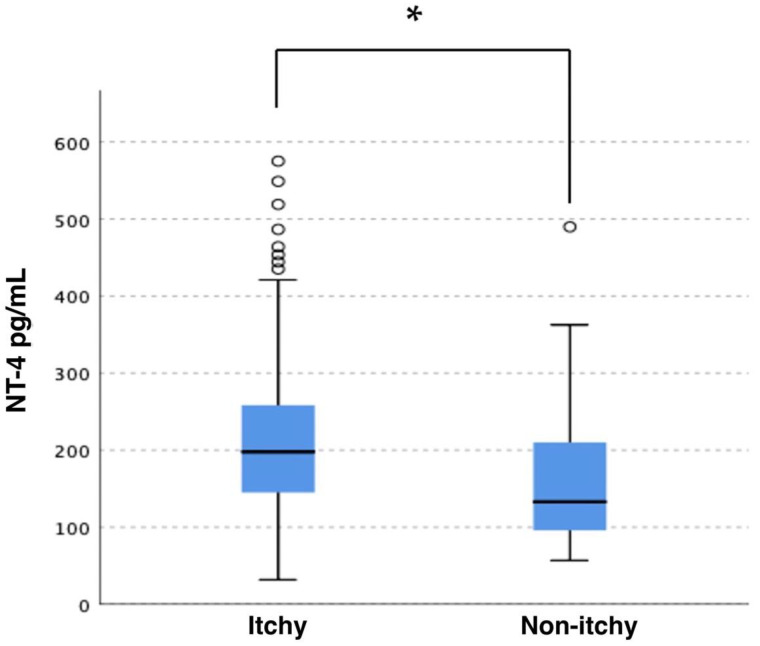
Serum level of NT-4 in groups of patients with pruritus and without pruritus. * *p* = 0.003.

The study has shown that patients undergoing dialysis with CKD-aP have statistically significantly higher levels of NT-4 compared to the non-pruritic group. The determined concentrations were 224.4 ± 128.8 pg/mL and 159.1 ± 90.0 pg/mL, respectively (*p* = 0.003). A comparison of NT-4 serum levels in both groups of patients is presented in Figure 2.

The serum level of BDNF in the pruritic group was 55.3 ± 66.0 pg/mL, while in the non-pruritic group it was 64.2 ± 62.7 pg/mL. The difference was not statistically significant. Moreover, no statistically significant differences and correlations were found between NT-4 nor BDNF concentrations and demographic data, including age, gender, duration of dialysis, type of access, or the cause of renal failure (detailed data not shown). The results of neurotrophin concentrations are summarized in Table 2.

### 3.3. Pruritus Assessment and Serum Levels of NT-4 and BDNF

In the group of patients undergoing dialysis with CKD-aP, the mean severity of pruritus on the 1–10 NRS scale was 4.9 ± 2.2 points. Most often, patients experienced moderate pruritus (48.4%). In turn, moderate to severe pruritus was reported by 70% of participants (Table 3). No statistically significant differences in NT-4 and BDNF serum concentrations were found between the four pruritus severity groups. Despite the higher concentration of NT-4 in this group of patients compared to the non-pruritic group, no correlation was found between the level of NT-4 nor BDNF and the severity of pruritus on the NRS (detailed data not shown). The mean UP-dial total score of itch in the studied group was assessed as 14.2 ± 9.8 points and did not correlate with both NT-4 and BDNF serum levels. Additionally, no significant relationships between ItchyQoL and serum concentrations of studied NTs were found (detailed data not shown).

## 4. Discussion

The role of neurotrophins in the proper functioning of the skin and maintaining its homeostasis has long been the subject of many studies. It has been known that NTs in the skin act as transmitters between skin cells and nerve endings in the dermis [31]. They are released by activated mast cells and eosinophils, as well as by keratinocytes and neuronal projections [20,32]. As previously mentioned, both BDNF and NT-4 bind to the TrkB receptor belonging to the tyrosine kinase family and the p75 neurotrophin receptor. In vivo studies in mice have shown that TrkB is present on the neurons innervating Ruffini and Meissner corpuscles, which are mechanoreceptors responsible for touch and stretching sensation. Furthermore, it has been noticed that increased TrkB expression leads to greater innervation of the skin and may be involved in the pathophysiology of some skin diseases [33].

The mechanism of action of NTs in the pathogenesis of skin diseases is still not fully understood. However, there are many studies investigating the contribution of these molecules to various, mainly pruritic skin conditions. In a clinical study by Raap et al. [34] performed in patients with atopic dermatitis (AD), the level of BDNF and the expression of the TrkB were measured. It has been noticed that AD patients have an increase in serum BDNF concentration and higher TrkB expression on eosinophils compared to people without atopic diseases. In addition, BDNF has been shown to inhibit eosinophil apoptosis and modulate eosinophil function, suggesting BDNF involvement in the pathophysiology of pruritic inflammatory skin diseases such as AD [34]. In several studies, it has also been observed that serum levels of BDNF correlate with the severity of AD in both children and adults [35,36]. A strong correlation was demonstrated between the BDNF concentration in the serum of children with AD and nocturnal scratching of the skin, which additionally confirms the role of this neurotrophin in the pathomechanism of pruritus [37]. Moreover, a significantly increased level of BDNF in the serum and altered skin was noticed in patients with chronic spontaneous urticaria [38]. Interestingly, in skin diseases such as vitiligo, psoriasis or acne vulgaris the level of BDNF is significantly lower compared to the control group [39,40,41,42]. However, the cause of this phenomenon has not been elucidated. A suggested hypothesis is chronic intense stress, often associated with the pathophysiology of certain dermatological conditions, which significantly reduces BDNF levels [43]. Interestingly, in another study on pruritus in psoriasis patients, no statistically significant differences in BDNF expression were observed between pruritic and non-pruritic patients [44].

The literature shows a large discrepancy in BDNF concentration in patients with CKD. It has been shown in numerous studies that the expression of BDNF is enhanced in patients with CKD or undergoing hemodialysis [9,45,46,47]. This may be related to the fact that this neurotrophin has been found to have a significant effect on glomerular development and function and is also involved in the repair of podocyte damage [45,46]. However, completely opposite results were obtained in the studies by Ortíz et al. [48] and Żołądź et al. [49] where the level of BDNF in CKD patients requiring hemodialysis was significantly lower compared to the healthy control group. In turn, Lee et al. [50] assessed the plasma level of BDNF and depressive symptoms in patients with CKD, but no differences in the concentration of BDNF were observed between patients with CKD and healthy subjects. Several studies have focused on studying BDNF levels in patients with CKD due to diabetic nephropathy. It has been found that in diabetic patients the plasma level of BDNF is significantly higher compared to patients with CKD but without diabetes [46]. However, our study did not show any correlation between BDNF levels and the cause of renal failure, including diabetic nephropathy. There is also no clear data on the association between BDNF level and gender. Our study did not show any difference between the concentration of this neutrophin and the gender of the patients. In the study by Marchelek-Myśliwiec et al. [51], in patients with chronic kidney disease, a higher concentration of this neutrophin was found in men, while in the study by Endlich et al. [46] BDNF concentration was almost twice as high in female as in male patients. Despite studies confirming the participation of BDNF in the pathogenesis of pruritic dermatological diseases and data suggesting an increased expression of this factor in patients with CKD, there is currently insufficient evidence in the literature to acknowledge the role of BDNF in the pathogenesis of CKD-aP. As in our study, Sorour et al. [9] did not show a statistically significant difference in BDNF concentration between the group of patients undergoing dialysis accompanied by pruritus compared to the group of patients undergoing dialysis without pruritus. There was also no correlation between the concentration of BDNF and the severity of itching [9].

In the case of NT-4, there are fewer publications assessing the level of this neurotrophin in dermatological diseases and conditions associated with pruritus. In vitro study by Grewe et al. [52] showed that activation of keratinocytes with interferon-gamma leads to an increase in NT-4 production. In turn, immunohistochemistry of human skin showed increased expression of NT-4 in irritated skin injected with gamma interferon. In addition, intense NT-4 staining in the biopsy of itchy lesions of AD patients was observed [52]. However, in a study by Chang et al. [53] in patients with psoriasis, NT-4 serum levels did not differ statistically between pruritic and non-pruritic patients [53]. So far, there is only a single publication examining the influence of NT-4 on the occurrence of CKD-aP. Sorour et al. [9] showed a significant statistical difference in NT-4 concentration between the groups of patients undergoing hemodialysis with and without pruritus. Moreover, it has been proved that the dependence on NT-4 concentration correlates positively with the severity of pruritus [9]. In our study, we also showed that the concentration of NT-4 is significantly higher in the group of dialysis patients with pruritus compared to those without pruritus. However, no positive correlation was found between the severity of the itch and the concentration of this neurotrophin in the patient’s serum.

Treatment of CKD-aP is a great challenge and despite many therapeutic options, including local and systemic treatments, many of them show insufficient anti-itching effects. A promising drug seems to be difelikefalin, which has recently been approved for the treatment of CKD-aP. However, as it is a new drug, data on its long-term effectiveness is limited [54]. Therefore, new possibilities are constantly searched for. Considering the possible involvement of NT-4 and BDNF in the pathomechanism of CKD-aP, further studies on the inhibition of neurotrophin-dependent activation of neuronal sensory pathways leading to pruritus are required. So far, no TrkB receptor antagonist has been identified that could be used in clinical trials in pruritic patients. In contrast, molecules that function as TrkB agonists that may be effective in the treatment of neurological and psychiatric diseases are currently being sought [55]. However, clinical trials with Pegkantratinib (CT327, SNA-120)—antagonist of other neutrophin receptors—TrkA in patients with pruritic skin disorders such as AD or psoriasis are ongoing or have already been completed [56,57]. A randomized phase II clinical trial has shown the efficacy of Pegkantratinib in reducing pruritus in patients with psoriasis [56]. Given the important role of neurotrophins in the proper functioning of the nervous system, inhibition of TrkB could be associated with numerous side effects. Therefore, studies on a TrkB antagonist that, like Pegcantratinib, would be applied topically seem to be the most warranted.

The limitation of the study is the assessment of only selected neurotrophins. The concentration of e.g., nerve growth factor was not evaluated in the study. Moreover, patients with CKD and other kidney replacement therapy, such as peritoneal dialysis, were not included in the study.

## 5. Conclusions

The results of the presented study indicate that in patients with end-stage renal disease undergoing hemodialysis, the occurrence of chronic pruritus correlates with elevated serum levels of NT-4. NT-4 may play an important role in the pathophysiology of pruritus in dialysis patients. More research is needed to understand the exact mechanism by which neurotrophins influence the occurrence of CKD-aP. The results of the study further confirm the importance of looking for substances that would inhibit neurotrophin-dependent interactions between the skin and the nervous system and could be used in the treatment of CKD-aP.

## Figures and Tables

**Table 1:** Characteristics of the study participants.

Parameter	Group A–Itchy	Group B–Non-Itchy	*p*-Value
All participants, n (%)	62 (100.0)	64 (100.0)	
-men/women	30 (48.4)/32 (51.6)	31 (48.4)/33 (51.6)	NS
Age, mean ± SD	61.1 ± 15.9	63.9 ± 15.6	NS
Duration of dialysis (months), mean ± SD	51.4 ± 44.5	46.3 ± 58.4	NS
Cause of CKD, n (%)			NS
-glomerulonephritis	8 (12.9)	17 (26.6)	
-diabetic nephropathy	11 (17.8)	13 (20.3)	
-ischaemic nephropathy	9 (14.5)	8 (12.5)	
-others	34 (54.8)	26 (40.6)	
Access, n (%)			*p* < 0.005
-tunneled internal jugular central venous catheter	30 (48.4)	14 (21.9)	
-arterio-venous fistulae	32 (51.6)	50 (78.1)	

NS—not statistically significant, SD—standard deviation.

**Table 2:** Plasma concentrations of NT-4 and BDNF in dialysis patients with (group A) and without (group B) pruritus.

Parameter.	Group A–Itchy			Group B–Non-Itchy			*p*-Value
	Mean ± SD	Median	Q1; Q3	Mean ± SD	Median	Q1; Q3	
NT-4 (pg/mL)	224.4 ± 128.8	197.7	144.5; 260.5	159.1 ± 90.0	133.0	95.4; 210.0	0.003
BDNF (pg/mL)	55.3 ± 66.0	31.2	13.1; 65.8	64.2 ± 62.7	52.9	16.3; 85.5	NS

N/A—not applicable, NS—not statistically significant, SD—standard deviation, Q1—first quartile (25th percentile), Q3—third quartile (75th percentile).

**Table 3:** Data on the severity of pruritus and quality of life in patients with CKD-aP.

Parameters	Result
NRS, mean ± SD, points	4.9 ± 2.2
Severity group based on NRS score, n (%)	
mild pruritus	16 (25.8)
moderate pruritus	30 (48.4)
severe pruritus	14 (22.6)
very severe pruritus	2 (3.2)
UP-Dial total, mean ± SD, points	14.2 ± 9.8
signs and symptoms domain	9.1 ± 4.5
sleep domain	2.8 ± 3.8
psychosocial domain	2.3 ± 2.9
ItchyQoL, mean ± SD, points	36.7 ± 13.7

NRS—numerical rating scale; SD—standard deviation.

## Data Availability

The datasets generated and analyzed in the current study are available from the corresponding author upon reasonable request. Acknowledgments: The authors would like to thank Marzena Lesik, a chair of the Dialysis Unit at the University Clinical Hospital in Opole, for their valuable help in collecting patients’ blood samples.

## Supplementary Materials

The following supporting information can be downloaded at: https://www.mdpi.com/article/10.3390/jcm11216292/s1, Figure S1: Polish version of the ItchyQoL questionnaire.
