# Supplementary material for: Elevated Level of Serum Neurotrophin-4, but Not of Brain-Derived Neurotrophic Factor, in Patients with Chronic Kidney Disease-Associated Pruritus
Source: J Clin Med. 2022 Oct 26;11(21):6292. doi: 10.3390/jcm11216292 (PMC9653946; doi:10.3390/jcm11216292)
Supplement: Supplementary file 1 [file jcm-11-06292-s001.zip › jcm-1969348-supplementary.pdf]

ItchyQoL

Poniższe pytania dotyczą Państwa odczuć związanych ze swędzącą chorobą skóry. Proszę zaznaczyć odpowiedź, która najdokładniej opisuje Państwa doznania	Jak często w ciągu ostatniego tygodnia poniższe stwierdzenia odnosiły się do Państwa sytuacji?				
	Nigdy	Rzadko	Czasami	Często	Cały czas
1. Zmiany skórne spowodowane swędzącą chorobą skóry krwawią	1	2	3	4	5
2. Zmiany skórne spowodowane swędzącą chorobą skóry bolą	1	2	3	4	5
3. Zmiany skórne spowodowane swędzącą chorobą skóry pieką i kłują	1	2	3	4	5
4. Mam blizny z powodu swędzącej choroby skóry	1	2	3	4	5
5. Muszę drapać się w miejscach swędzenia skóry	1	2	3	4	5
6. Zmiany temperatury lub pór roku nasilają odczuwaną przeze mnie swędzącą chorobę skóry	1	2	3	4	5
7. Wydaje mnóstwo pieniędzy na leczenie swędzącej choroby skóry	1	2	3	4	5
8. Swędząca choroba skóry sprawa, że ciężko jest mi pracować lub robić to, co lubię	1	2	3	4	5
9. Swędząca choroba skóry wpływa na moje kontakty z innymi ludźmi (np. rodzina, przyjaciele, bliskie relacje itd.)	1	2	3	4	5
10. Swędząca choroba skóry źle wpływa na mój sen	1	2	3	4	5
11. Swędząca choroba skóry często nie pozwala mi się skoncentrować	1	2	3	4	5
12. Swędząca choroba skóry ogranicza rodzaje ubrań, które noszę	1	2	3	4	5
13. Swędząca choroba skóry zmusza mnie do kupowania specjalnych środków do prania i płynów kosmetycznych	1	2	3	4	5
14. Frustruję się z powodu choroby skóry	1	2	3	4	5
15. Wstydzę się z powodu choroby skóry	1	2	3	4	5
16. Świąd skóry doprowadza mnie do szału	1	2	3	4	5
17. Swędząca choroba skóry sprawa, że jestem zły/a lub łatwo się denerwuję	1	2	3	4	5
18. Swędząca choroba skóry sprawia, że czuję się smutny/a lub popadam w depresję	1	2	3	4	5
19. Martwię się tym, co myślą inni ludzie w związku ze stanem choroby skóry	1	2	3	4	5
20. Martwię się, że swędzenie będzie trwać wiecznie	1	2	3	4	5
21. Z powodu choroby skóry czuję się zbytnio skupiony/a na stanie swojej skóry	1	2	3	4	5
22. Z powodu stanu choroby skóry zmieniła się moja osobowość	1	2	3	4	5

SUMA PUNKTÓW: ………………………………

Figure S1 Polish version of the ItchyQoL questionnaire
